# Analysis of Factors Affecting Pregnancy Rate After Artificial Insemination in Lusitano Broodmares

**DOI:** 10.3390/ani16213528

**Published:** 2026-11-07

**Authors:** Inês B. Carvalho, Luis T. Gama, Helena Guimarães, Madalena Cardoso, Maria José Correia, Madalena Roquete, Ana Rita Gonçalves, António Pinto, Joana Rodrigues, Elisa Bettencourt

**Affiliations:** 1 MED Mediterranean Institute for Agriculture, Environment and Development & CHANGE Global Change and Sustainability Institute, Institute for Advanced Studies and Research, Universidade de Évora, Largo dos Colegiais 2, 7004 Évora, Portugal; 2 CIISA—Centre for Interdisciplinary Research in Animal Health, Faculty of Veterinary Medicine, Associate Laboratory for Animal and Veterinary Sciences (AL4AnimalS), Universidade de Lisbon, 1300 Lisbon, Portugal; 3 Alter Clinical Unit, Veterinary Teaching Hospital of Évora University, Tapada do Arneiro, 7440 Alter do Chão, Portugal; 4 Animal Protection Division of Alentejo—North, Alentejo Regional Directorate for Food and Veterinary Services, 7000 Évora, Portugal; 5 VetAlter, 7440 Alter do Chão, Portugal; 6 Coudelaria de Alter, Companhia das Lezírias, Tapada do Arneiro, 7440 Alter do Chão, Portugal; 7 Veterinary Medicine Military Unit, Portuguese Army, 2640 Mafra, Portugal; 8 Department of Veterinary Medicine, School of Science and Technology, University of Évora, 7000 Évora, Portugal

**Keywords:** reproduction, fertility, stud management, mare, breeding season

## Abstract

Artificial insemination (AI) is widely used in the horse breeding industry, but its success is often limited by different factors. Our objective was to assess the importance of various factors affecting fertility after AI, using field data collected in 125 mares over a period of 20 years, corresponding to a total of 860 mare-year combinations. The criteria defined for measuring fertility were: (1) pregnancy rate per breeding season; (2) pregnancy diagnosed at the 14th day on the first cycle and (3) number of cycles needed for a mare to become pregnant. Statistical analyses were performed using the GLIMMIX procedure of SAS, with a binary logit model with repeated records. The overall pregnancy rate per breeding season (PRBS) was 0.813 and at 14 days on the first cycle (PR1C) it was 0.546. The PRBS was higher when insemination was practiced early in the breeding season (*p* < 0.001) and declined with increasing mare age (*p* < 0.001) as well as with stallion age (*p* = 0.013). Mares presenting uterine pre-AI oedema had higher fertility at PR1C (*p* = 0.037). The mean number of oestrous cycles for a confirmed pregnancy was 1.82 ± 1.01 and, among others, age impacted this result adversely. In conclusion, our results suggest that some identified key factors can be used by owners, farm managers and veterinarians to improve mare reproductive performance.

## Simple Summary

The success of artificial insemination in horses may be influenced by a wide range of factors. The aim of this study was to assess the importance of various factors affecting fertility in an insemination programme featuring Lusitano broodmares. The overall fertility after artificial insemination was 81.28%, and a higher pregnancy rate was achieved in mares bred earlier in the season while an increased age of the mare or stallion produced a marked deleterious effect on fertility. The repeatability of mare fertility after AI was low, indicating that the consistency of repeated pregnancy records in a given mare is not high. Our results suggest that some identified key factors can be used by all involved in equine reproduction to improve reproductive performance in a stud farm. Fertility is multifactorial and it is nowadays acknowledged that management can influence fertility more directly and profoundly than has previously been accounted for.

## 1. Introduction

The main goal in horse production is to ensure a healthy foal produced every year per mare bred [1] and artificial insemination (AI) is widely used in the horse breeding industry [2]. Reproductive performance outcomes (i.e., pregnancy rate, pregnancy loss rate and live foal rate) are usually measured per cycle or per season [3]. The per cycle rates assess the efficiency, accounting for how many mating attempts are required to achieve a positive pregnancy result. On the other hand, the per reproductive season rates evaluate the overall effectiveness of achieving the intended reproductive outcome. In addition to overall effectiveness, breeders should be concerned with the efficiency of achieving pregnancy to maintain success over time because, if mares foal later in each successive year, they may end up foregoing a breeding season [3]. A number of studies on reproductive performance have been conducted, reporting a range of 58–68% [3,4,5,6] for pregnancy rate per cycle and 79–95% per season [2,3,4].

The success of reproductive performance in horses may be influenced by a wide range of factors. The elucidation of these factors is of paramount importance to improve the success of breeding horses, since their management will allow reproductive success to improve in many cases. In recent years, equine reproduction has improved due to enhancements in stallion and mare management and veterinary care [1,3]. Likewise, mare owners have become increasingly concerned with the costs of producing a healthy foal, which include, for example, management of the mare’s reproductive cycle, charges during pregnancy and stallion stud fee [3]. Both mare and stallion influences have been shown to affect reproductive performance in breeding programmes. For example, age of the mare has a strong influence on fertility [1,2,3,4,5,6,7,8], because of the reduced pregnancy rate per cycle and higher pregnancy loss rate in older mares [2]. In addition, factors such as foal heat [1,4,5], reproductive status of the mare (i.e., if the mare was classified as maiden, foaled or barren) [1,3], month of AI [1,4], inbreeding of the mare and stallion [7,9], stallion age [7], frequency of usage of the stallion and type of semen used [2] have been shown to be related to reproductive success [5].

The aim of this retrospective study was to evaluate some factors that affect the reproductive success of an insemination programme applied over a period of several years in Lusitano horses. The studied factors were age of mare and stallion, coefficient of inbreeding of mare and stallion, mare reproductive status, month of AI and year of breeding. In a second study, fertility at 14 days on the first cycle was analysed, considering the same factors, plus the effect of the presence of uterine oedema, occurrence of endometritis and type of semen used (fresh, refrigerated or frozen).

## 2. Materials and Methods

### 2.1. Ethical Approval

Owner consent for the use of data from clinical records for research purposes was obtained from the responsible of the stud prior to inclusion in this study.

### 2.2. Data Collection

The data analysed were reproductive records of Lusitano mares obtained from an artificial insemination (AI) programme of a Pure Breed Lusitano stud farm over a period of 20 years.

Two studies were carried out, considering different types of information.

*Study 1*

**Figure 1:**
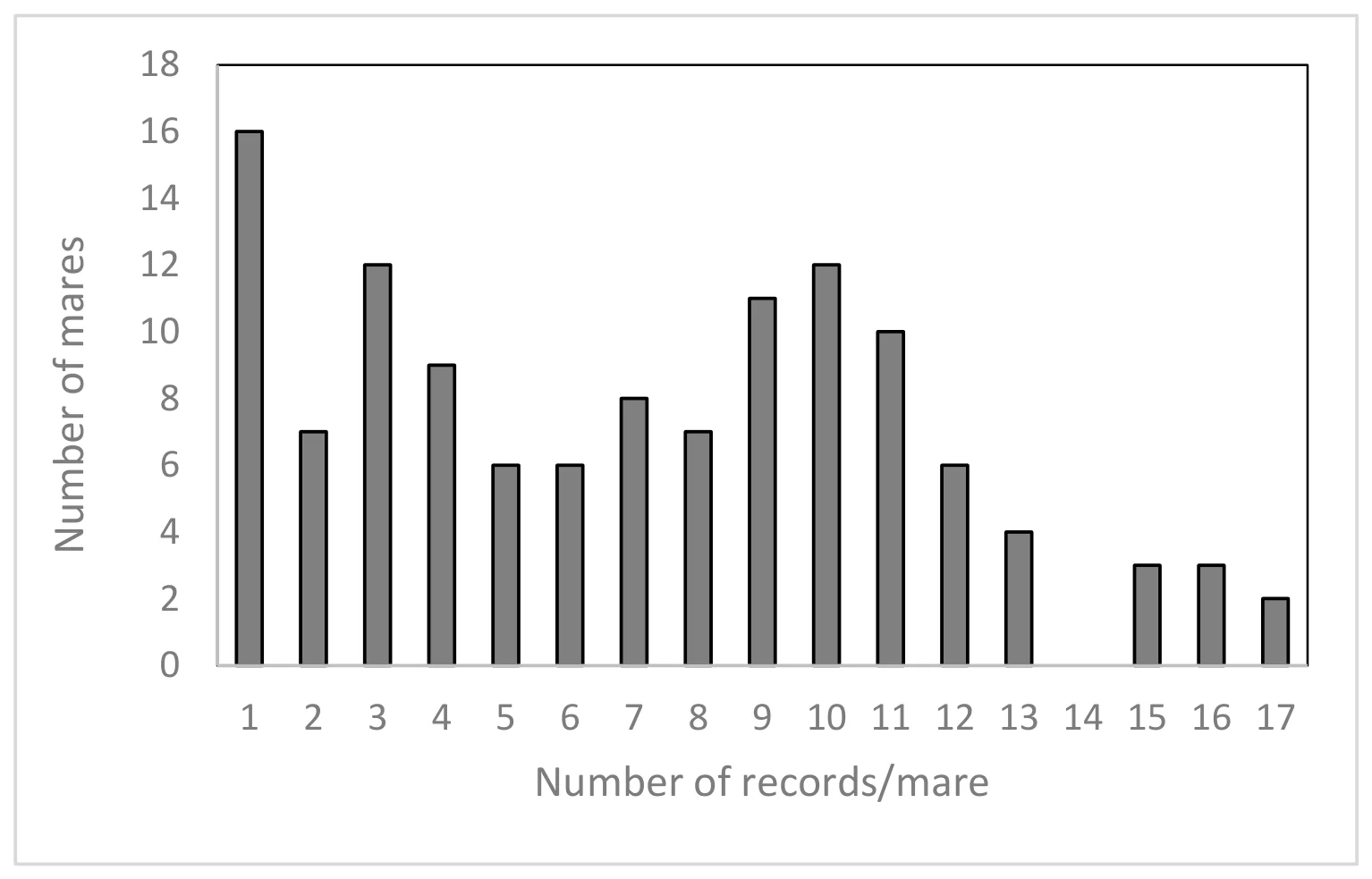
Distribution of the number of artificial insemination records per mare in the data set analysed.

In the first study, reproductive data collected after artificial insemination in 125 mares over a period of 20 years, corresponding to a total of 860 mare-year combinations, was used to investigate the effect of various factors on fertility per reproductive season. There was a mean of 7.05 ± 4.36 records collected per mare, with the distribution shown in Figure 1.

Pregnancy rate per year was defined as the number of pregnant mares at the end of the breeding season relative to the number of mares inseminated and present at the time of foaling.

Pregnancy rate was analysed by logistic regression with a binary logit model, considering repeated records of mares. Fertility of a mare in a given year was coded as 0/1, and the following factors and corresponding statistics or categories were considered as potentially affecting the success of AI:

- Years of breeding (20 years).
- Age of mares (10.79 ± 4.85 years, range of 4 to 29 years).
- Coefficient of inbreeding of the mare (mean 9.81 ± 5.93%, range 0 to 28.14%).
- Mare reproductive status: maiden (never mated), foaled (foaling in that breeding season), or barren (mated without a pregnancy at the end of the previous season or deliberately not mated).
- Months of AI: February to June.
- Age of stallion (mean of 13.17 ± 5.89 years, range of 4 to 31 years).
- Coefficient of inbreeding of the stallion (mean of 10.47 ± 4.81%, range of 0 to 22.5%).

*Study 2*

**Figure 2:**
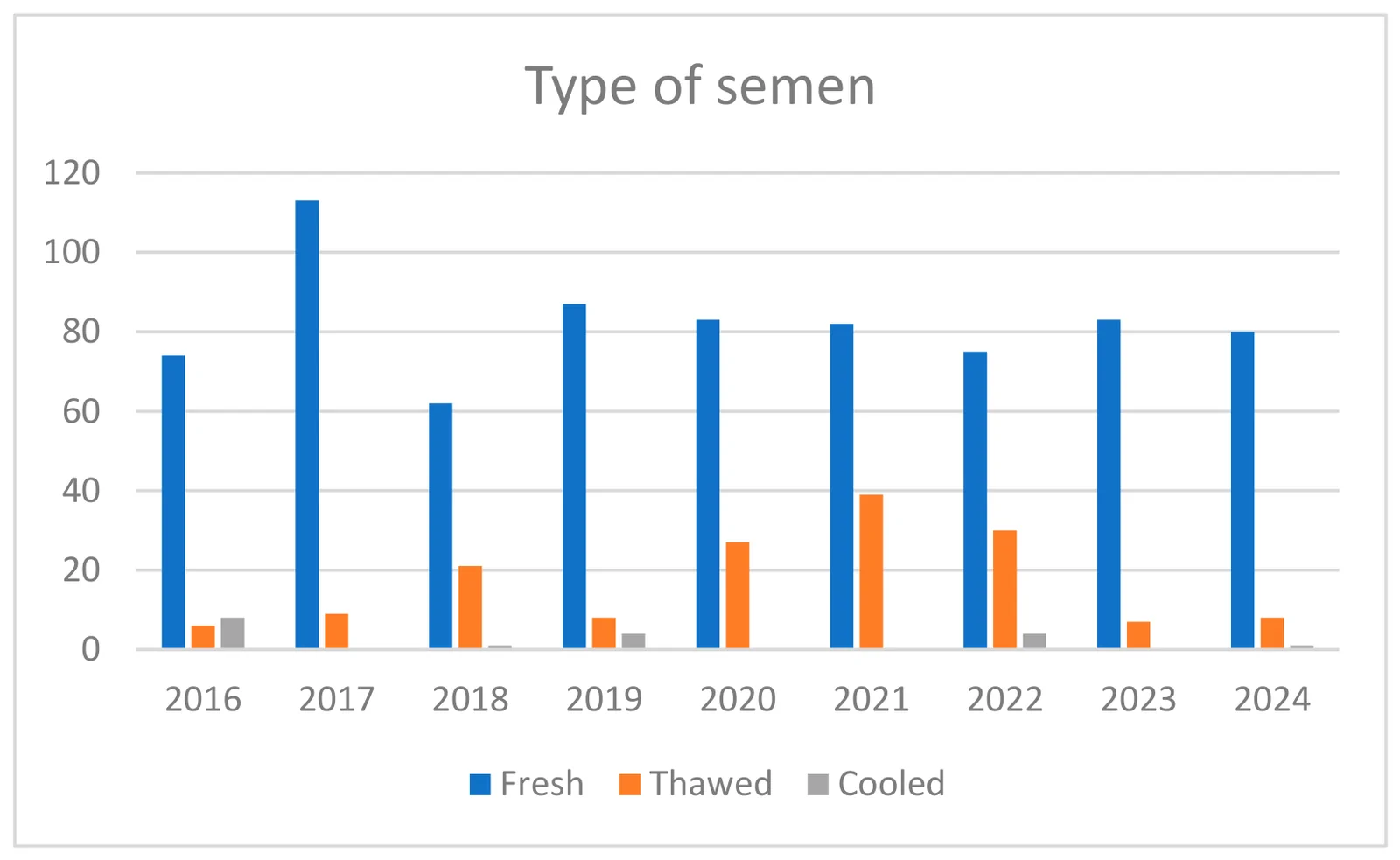
Distribution of the yearly proportions of the different types of semen used.

A second study was conducted with data collected in the period from 2016 to 2024, where, in addition to common breeding records as those in study 1, detailed ultrasound information was available for 473 insemination records in 98 mares. In this study, we assessed fertility at 14 days on the first cycle, as well as the number of oestrous cycles required to achieve a pregnancy. Fertility at 14 days on the 1st cycle was coded as 0/1 for each mare, and the ratio of pregnant mares at 14 days on the 1st cycle relative to the number of inseminated mares was computed. The factors considered in the logistic regression analysis of fertility at 14 days on the 1st cycle were the same as those listed for pregnancy rate per year, plus the presence of oedema pre-AI (score: 0: no oedema, 1: mild oedema, 2: moderate oedema, 3: severe oedema [10,11]), occurrence of endometritis and type of semen used (fresh, refrigerated or frozen). The yearly proportions of the different types of semen used are shown in Figure 2.

To minimise subjectivity in uterine oedema scoring, reproductive examinations were routinely performed jointly by the attending veterinary team to standardise evaluation criteria. Furthermore, all ultrasonographic examinations were conducted using a single ultrasound unit with fixed depth and gain parameters, thereby maximising inter-observer reliability.

In the analysis of the number of cycles needed to obtain a confirmed pregnancy, records on mares that conceived were considered, and the factors studied were year of breeding, month of AI, mare reproductive status, age of the mare and inbreeding coefficient of the mare.

### 2.3. Reproductive Management

Each year, the reproductive season occurred between February and June. Follicular development was monitored using sequential transrectal ultrasonography from the onset of oestrous (maiden and barren) or 8 days post foaling (foaled mares). When a follicle with mean diameter greater than 35 mm was detected, ovulation was induced (hCG or synthetic analogues of GnRH, triptorelin). Mares were inseminated with either fresh, cooled or thawed frozen semen from different available stallions. Insemination with fresh and cooled semen was performed on the day after induction of ovulation while mares managed for the use of frozen semen were checked every 6 h to be inseminated post-ovulation (maximum of 6 h after ovulation). At the time of insemination, the mare was placed in standing stocks and the tail was wrapped up. The perineum was scrubbed with chlorhexidine, rinsed with tap water and thoroughly dried with clean disposable paper towels. Semen was deposited either in the uterine body (fresh and cooled semen, minimum of 500× 10^6^ mobile spermatozoa) or deep in the uterine horn ipsilateral to the confirmed ovulation, after the straws were thawed in a water bath and dried (frozen semen, minimum of 300× 10^6^ mobile spermatozoa). Pregnancy status was determined using transrectal ultrasonography on Day 14 and Day 45 after ovulation. In study 2, ultrasonographic evaluation before AI also included assessment of the presence and maximal height (cm) of intrauterine fluid measured in the uterine body and endometrial oedema. The presence of endometritis was considered when uterine fluid and severe oedema occurred, and confirmed by laboratory analyses, such as cytology and microbiology.

### 2.4. Statistical Analysis

Statistical analyses were performed with SAS software version 9.4 (SAS Institute, Cary, NC, USA). The primary response variable in study 1 was the occurrence of pregnancy at the end of the breeding season (PRBS), while in study 2 it was the occurrence of pregnancy at 14 days on the first cycle (PR1C). In both cases, the response variables were categorical and logistic regression was used to investigate the significance of the various factors considered. Moreover, repeated records were collected on the same mare, and this should be taken into account in the statistical analyses.

In the first study, data were analysed with the GLIMMIX procedure of SAS, using a binary logit model with repeated records. In this study, fertility after AI was considered to be a function of the fixed effects of year of breeding, mare reproductive status, and month of insemination, while the level of inbreeding of the mare and stallion as well as their age were considered as linear covariates. As the data included repeated mare records, a random intercept component was included in the binary logit model to account for variability among mares, and thus for the possible association between records collected in the same mare, assuming a compound symmetry structure.

In the second study, the pregnancy rate at 14 days on the first cycle was also analysed with the GLIMMIX procedure of SAS, using a binary logit model with repeated records. In this case, the model was similar to that used for pregnancy rate per season, but the fixed factors considered also included the occurrence of endometritis, pre-AI endometrial oedema and type of semen used.

In the analysis with GLIMMIX, the estimated covariance between records on the same mare, and thus the random-intercept variance between animals, can be used to assess the tendency for a mare to have consistently a higher or lower fertility. Because this is a binary logit model, the residual variance (*σ*^2^) can be computed as [12]

$$\sigma^{2}=\frac{\pi^{2}}{3}\approx 3.2899$$

Therefore, the estimated correlation between repeated records collected on the same animal can be computed as

$$\hat{\rho }=\frac{{\hat{\sigma }}_{animal}^{2}}{{\hat{\sigma }}_{animal}^{2}+3.2899}$$

The third analysis considered the number of cycles needed for a mare to become pregnant, and a mixed model with repeated records was used in the GLIMMIX procedure of SAS. The fixed factors included in the model were year of breeding, month of AI and reproductive status, plus the covariates age and inbreeding coefficient of the mare. As for the other analyses, a random intercept component was included in the mixed model to account for variability among mares.

## 3. Results

*Study 1*

A total of 125 mares that underwent AI were followed for a total of 20 years, resulting in 860 mare-year records. The number of inseminations performed per year ranged from 20 to 61 (Table 1), and increased over the period analysed. The majority of the inseminations took place between March and May, with a smaller number in June and February (Table 1).

The overall mean fertility per season after AI was 81.28%, and the statistical significance of the various factors considered in logistic regression is summarised in Table 2. The factors with the more significant effect on fertility were month of AI (*p* < 0.001), age of mare (*p* < 0.001), and age of stallion (*p* = 0.013), while year of breeding approached significance (*p* < 0.10). On the other hand, the reproductive status of the mare and the level of inbreeding of the stallion and of the mare did not have a significant effect on pregnancy per season (*p* > 0.10).

The likelihood of pregnancy and the odds ratios for the various months of insemination are shown in Table 3. The mean pregnancy rate and the corresponding odds ratio were similar (*p* > 0.05) for inseminations practiced between February and April, with fertility of about 0.97. A significant decline in fertility to about 0.89 was observed in inseminations performed in May (*p* < 0.05), and even further in June inseminations (*p* < 0.05), where the odds ratio was 0.7566, with a likelihood of pregnancy of about 0.68.

A steady decline in pregnancy rate was observed as age increased in both mares and stallions (Table 4), with a regression coefficient on age of about −0.12/year for mares and −0.05/year for stallions.

In our analysis of pregnancy rate per season with GLIMMIX, the variance between animals, estimated by compound symmetry from the covariance between records of the same animal, was 0.2656. Therefore, the estimated repeatability of the likelihood to become pregnant in a given year after AI, measured on the underlying logit scale, is:

$$\hat{\rho }=\frac{0.2656}{0.2656+3.2899}=0.075$$

**Figure 3:**
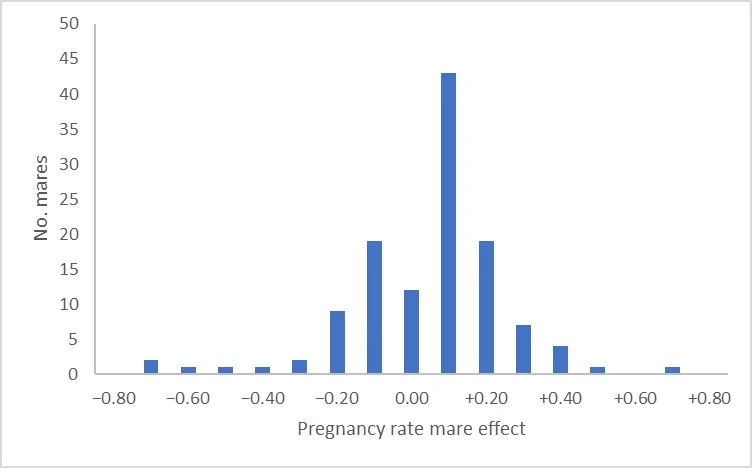
Distribution of the mare effects for pregnancy rate in logit model regression analysis of pregnancy rate per season.

The distribution of the individual estimates of mare permanent effects (Figure 3) indicates that the variability among mares in their likelihood to become pregnant is important. Still, there is no indication of a meaningful incidence of repeat breeders in this stud, as revealed by the relatively small proportion of animals with extreme values on the left tail of the distribution.

*Study 2*

The mean PR1C was 54.6%. Among the factors considered as possibly influencing PR1C (Table 5), a significant effect (*p* < 0.05) was found for age of stallion and oedema score before AI, and it approached significance (*p* = 0.062) for stallion inbreeding. The remaining factors considered did not have a significant effect on PR1C (*p* > 0.10).

The regression coefficients for the significant factors (Table 6) indicate that PR1C declined by about 0.035 when sire age increased by 1 year, and by about 0.038 when sire inbreeding increased by 1%. On the other hand, a higher degree of pre-IA endometrial oedema was associated with increased PR1C.

In the binary logit-model regression analysis of pregnancy diagnosis on the 1st cycle at 14 days post-AI with GLIMMIX, the estimated variance between animals was 0.00359, which corresponds to an estimated repeatability of only 0.001 for this trait. This indicates that the permanent effect of the mare on PR1C is essentially negligible.

A total of 911 oestrous cycles were followed during the 9-year period throughout February to June. Overall, the mean number of oestrous cycles for a confirmed pregnancy was 1.824 ± 1.009.

In the analysis of the number of cycles needed to obtain a confirmed pregnancy using a mixed model with repeated records, all the factors considered had a significant effect (*p* < 0.06), with the exception of reproductive status in the previous year (Table 7).

**Figure 4:**
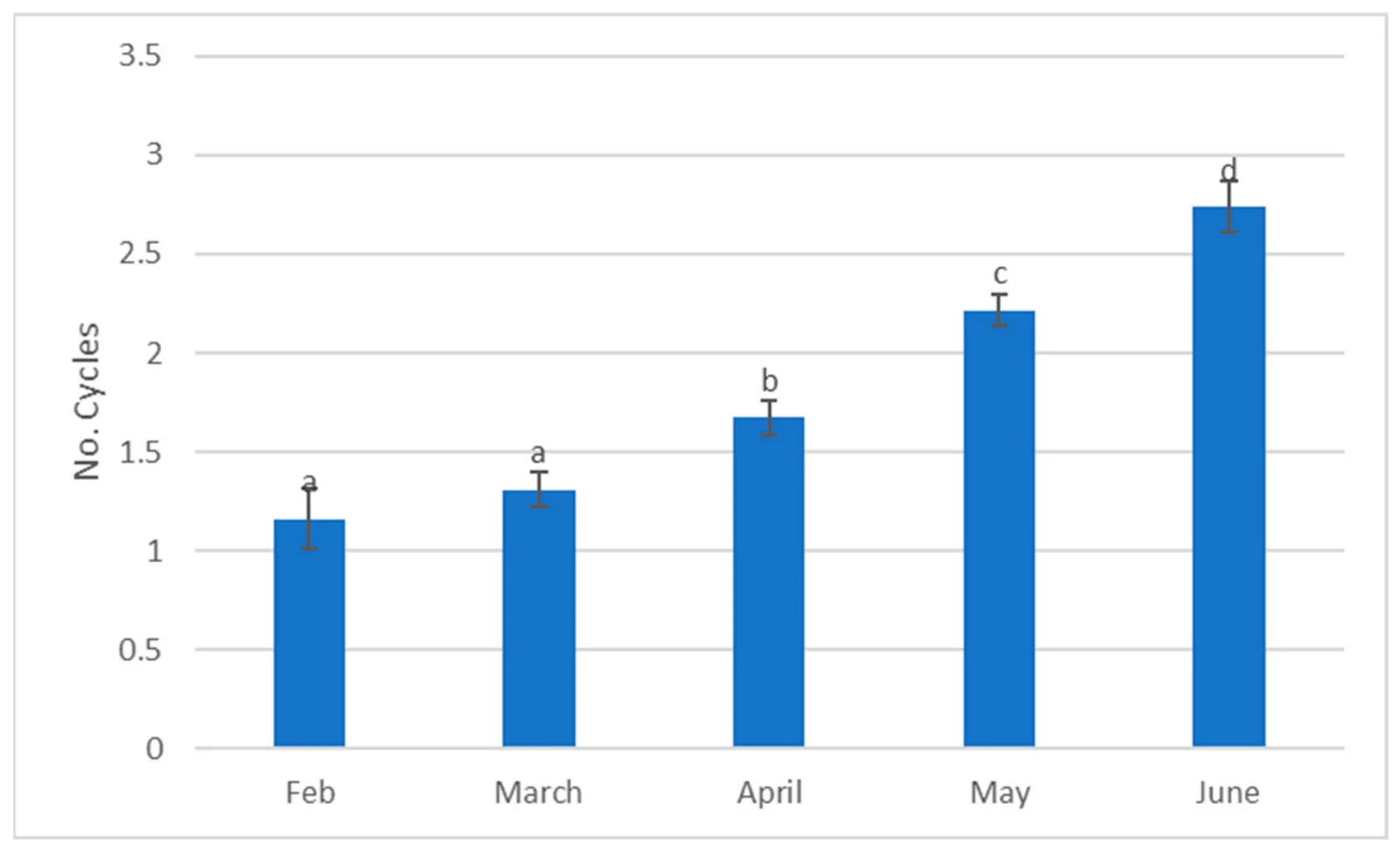
Least squares means and corresponding standard error for number of cycles to achieve a confirmed pregnancy after artificial insemination in Lusitano horses, by month of fertile AI. Means with different superscripts differ (*p* < 0.05).

The mean number of cycles for conception per month of insemination is in Figure 4. On average, in the early stages of the breeding season fewer cycles are needed for a confirmed pregnancy (mean of 1.17 in February and 1.33 in March) but, as the season progresses, more cycles were followed before a successful pregnancy was achieved to reach 2.74 cycles in June.

The estimated regression coefficient of number of cycles to achieve a confirmed pregnancy on the age of mare indicates that a significant increase occurs in older mares, with a mean increase of 0.022 cycles/year of age (Table 8). On the other hand, there was a slight decrease related to inbreeding on the number of cycles needed to achieve a confirmed pregnancy, which on average declined by about −0.017 cycles/1%F (Table 8). Even though the magnitude of this effect is not large, it would indicate that more inbred mares would require fewer inseminations to get pregnant.

The mixed model analysis of number of cycles to obtain a pregnancy using GLIMMIX resulted in an estimate of 0.03565 for the covariance between records collected on the same mare and 0.7399 for the residual variance. Therefore, the estimated repeatability of the number of cycles needed to achieve a pregnancy is 0.047.

## 4. Discussion

In this retrospective study, we have used field data to evaluate different factors contributing to the reproductive success of an artificial insemination programme in Purebred Lusitano mares. Knowing the impact of the different factors in reproductive success will allow devising a breeding programme to improve equine fertility. We have assessed different responses (PRBS, PR1C and number of cycles needed to get pregnant) to acquire detailed information on the conditions investigated, not just in terms of effectiveness in getting a mare pregnant but also its efficiency. For example, the response variable ‘14th day pregnancy rate of the 1st cycle that a mare was followed’ (PR1C) will add valuable information on efficiency, since the mares that get pregnant at the first cycle will be inseminated only once, reducing the number of cycles per pregnancy. In this sense, it gives us evidence on the immediate factors that can be detrimental to the probability of the mare getting pregnant, but not to the probability of foaling.

The first part of our study included information from a period of over 20 years, focusing on the factors that affected PRBS (study 1). The overall pregnancy rate observed in this study (81.28%) is similar to that reported in studies on Norwegian Coldblooded trotter mares [2] and Standardbred mares [8], and compares favourably with other studies [5,6], likely reflecting good reproductive management [1,8]. In the second study, more information was available, which allowed a comprehensive investigation of additional factors associated with fertility, including PR1C, as well as the number of reproductive cycles needed to achieve pregnancy.

Fertility per season encompasses a multitude of factors impacting not only conception but also maintenance of gestation; it therefore evaluates the overall effectiveness of achieving the reproductive outcome [3]. For instance, ‘problem mares’ may be unlikely to get pregnant and maintain the pregnancy during consecutive cycles, and this will be reflected in these results.

When evaluating PRBS, the factors assessed in our study with a more meaningful effect were mare age, month of AI, and stallion’s age, while year of breeding approached significance.

Age of the mare is a common denominator in both studies when considering PRBS, PR1C and number of cycles required for pregnancy. Consistent with other studies, fertility was reduced with increasing age of the mare, which is often considered the factor with the most markedly negative effect on fertility [1,3,4,6]. Aged mares may have lower pregnancy rates, require more matings per conception and also the use of uterine therapy [4], which possibly reflects an impaired uterine environment and poor oocyte quality, both jeopardising embryo development and survival [4,13]. On the one hand, the progression of degenerative endometrial changes observed in older mares causes decreased endometrial quality and increased susceptibility to infection [4,14]. Plus, the presence of excessive uterine fluid retention is promoted by poor perineal conformation and a ‘pendulous’ uterus, conditions also associated with old age [15]. The overall impact that these age-derived changes have on the endometrial environment goes beyond the initial impact on early pregnancy, with studies showing a higher incidence of pregnancy loss in older mares [1,7]. On the other hand, another possible explanation for the impact of age is the compromised oocyte quality with advanced maternal age, most widely documented in women but also in the mare. Age-associated differences in oocytes include altered morphology, gene expression and developmental potential. The oocytes accumulate components before maturation that are essential for normal zygote and embryo development. In vivo studies support a high early embryo loss rate in older mares, with delayed development and more morphological abnormalities. Additionally, albeit not estimated in our study, the literature shows that one unique feature of equine reproduction is that performance parameters can be measured in offspring from mares of different ages, notably with offspring race performance markers declining for mares older than 10 years, hence with oocyte quality having long-lasting impact with potential alterations in progeny [13]. Future studies targeting this parameter would be of the utmost relevance for the equine industry.

Regarding age as a factor in the stallion, it impacted both PRBS and PR1C. Older stallions tend to have lower foaling rates than the younger ones, but this downward effect on male fertility with increasing age does not occur as rapidly as that of females [7]. In stallions, the effect of age may be explained by differences in sperm production. In Lusitano horses, semen concentration, an important parameter to evaluate semen quality, declines linearly as stallions age. Volume and motility had a quadratic relationship with stallion age, meaning lower values were found in younger and older horses [9]. In our study, and common practice in equine reproduction, the number of motile spermatozoa inseminated is standardised before AI; therefore it is reasonable to expect a less pronounced effect of stallion age.

The month of AI plays a significant role in fertility per season, usually with a higher fertility rate in mares bred earlier in the season. This could come for a number of reasons, such as temperature, nutrition, health and breeding opportunities. The lowest fertility per season was observed in June, which could be explained by the high temperatures in southern Portugal at this time of the year. In cattle, thermal stress has a well-documented detrimental effect on reproductive performance and a recent study in mares found out that high ambient temperature and humidity is associated with early embryonic loss after embryo transfer [16]. In our study, pregnancy rates kept relatively steady during the season until May and June when they dropped considerably. A prior study focused on Lusitano broodmares demonstrated that their reproductive efficiency is influenced by nutritional status and energy balance, revealing a pronounced effect of nutritional status at conception on fertility of mares. Mares increasing their body condition at conception present higher fertility [17]. Like other horse breeds in southern Europe, Lusitano horses are mainly raised on extensive production systems where pasture represents the main part of mares and foals’ diet and its availability are influenced by the time of the year. Notably, grass availability and quality considerably decrease after May in Portugal. In this sense, it does not come as a surprise that a relationship with the nutritional status of the mare, associated with pasture availability, may also justify the influence of the month of AI in our study. Additionally, in our study, the greatest number of mares cycling occurred during the months of April and May (Table 1). These data agrees with earlier work conducted in the United Kingdom showing the greatest number of coverings occurred during April [1]. These months correspond to the increased temperatures of late spring/early summer and a gradual decrease in nutritional support to broodmares extensively bred in southern Europe, combining two major drivers of decreased fertility. One study shows, despite no statistically significant effect of month of covering, a numerically lowered pregnancy rate experienced by mares covered later in the season [1]. On the one hand, this may reflect the population of potential subfertile mares that had taken longer to conceive within the breeding season or in previous seasons and are now part of the group foaling and breeding later in the following season again, in comparison to the high fertility of early cycling mares and foaling mares that had conceived without difficulty in the previous year [4]. On the other hand, mares bred late in the breeding season are also in disadvantage due to fewer breeding opportunities, hence compromising their pregnancy rate at the end of the reproductive season. The industry demands early born foals and later in the breeding season mares are potentially left non-pregnant so they can conceive early in the following year [8].

In the present study, significant inter-annual variations in fertility were observed. In equine species, yearly fluctuations in reproductive efficiency are frequently driven by climatic variations—such as rainfall patterns and ambient temperature—which dictate forage availability, pasture quality, and mare body condition score [17]. These nutritional oscillations modulate permissive metabolic signals, including leptin and IGF-1, which directly influence GnRH secretion and per cycle conception rates [18]. However, environmental and climatic parameters were not systematically recorded during the study period. Consequently, while inter-annual environmental and nutritional shifts offer a plausible explanation for the observed year effect, their specific contribution cannot be definitively confirmed in our dataset, highlighting the need for future studies integrating local agro-meteorological tracking.

The estimate of 0.075 for the correlation between pregnancy rate and repeated records can be interpreted as the repeatability of fertility after AI in mares. This indicates that, after accounting for the various fixed effects in the model, nearly 8% of the variation in pregnancy rate measured on the underlying logit scale can be attributed to consistent differences among individuals in their repeated binary records. This estimate is in line with the results reported by Sairanen et al. (2009) for measures of reproductive success in Finnish mares [7].

The evaluation of further individual factors that displayed significance on PR1C (the above-mentioned age of the stallion, as well as uterine oedema pre-AI) allowed the best evaluation of the parameters affecting reproductive efficiency. The pregnancy rate in this instance was of 54.6%, a value lower than the per season estimate, which reflects that not all mares get pregnant at the first cycle.

Uterine oedema score pre-AI was directly related to fertility, with higher fertility rates in mares with more oedema before insemination. Likewise, a previous study featuring Lusitano mares pointed out that uterine oedema correlated positively with the likelihood of a successful pregnancy [19]. Under oestrogen influence, prior to ovulation, prominent endometrial oedema is physiologic and can be used as a management tool for monitoring progress towards ovulation [10,19]. However, another study reports that the degree of uterine oedema does not influence pregnancy rate [10].

Stallion inbreeding approached significance, with the regression coefficient indicating that PR1C declines by about 0.038 when sire inbreeding increases by 1%. A recent study with Lusitano stallions [9] assessed the effect of inbreeding on semen characteristics and concluded that the individual level of inbreeding is an important factor negatively influencing the quality of semen. It demonstrated a significant negative effect of inbreeding on semen concentration, motility, total number of spermatozoa and total number of motile spermatozoa, the latter declining by about 1.5% per 1% increase in inbreeding of the stallion [9], which may have important consequences in male fertility. In our study, the mean inbreeding of stallions was 10.47 ± 4.81%, so a decline in fertility could possibly be anticipated. However, in AI the preparation of the semen doses to be applied is standardised (minimum of 500 × 10^6^ motile spermatozoa), and so the number of live spermatozoa actually inseminated should be uniform and may cancel a possible effect of stallion inbreeding on mare fertility. Both elements may explain our finding.

The number of reproductive cycles followed per mare each year is relevant for the efficiency of reproductive control not only from an economic point of view but also for mare reproductive health, being well known that mares inseminated more times are more prone to develop chronic endometritis as it can be triggered by exposure to semen [14]. As mentioned, the PRBS is high but must be evaluated with relation to the number of cycles followed, being different a mare that gets pregnant with one cycle compared to a mare that takes 3 or 4 cycles to get pregnant. On average, each mare was followed for 1.824 ± 1.009 oestrous cycles per confirmed pregnancy throughout our study. Multiple matings during the season may indicate fertility problems with the mare, stallion or both [3]. As expected, in the analysis of factors impacting the number of cycles to obtain a confirmed pregnancy, breeding early on the breeding season and breeding young mares require less number of cycles to confirm a pregnancy, further strengthening the motifs above-mentioned. Indeed, to achieve pregnancy, the mean number of cycles more than doubles from February to June and mares require 0.022 more cycles as they age yearly. On the other hand, mares with a higher level of inbreeding required fewer inseminations to get pregnant. Although inbreeding is typically associated with reduced reproductive performance in the literature [7], the observed decrease in the number of cycles per pregnant mare with higher inbreeding levels can be primarily attributed to a possible bias inherent to this metric. In breeding management, subfertile or non-conceiving inbred mares are frequently removed from the breeding herd after early unsuccessful attempts, leaving only highly fertile individuals within the subset of mares that successfully achieve pregnancy [20]. Furthermore, it can be anticipated that high-value inbred mares often receive intensified veterinary management and optimised oestrus monitoring, which maximises per-cycle conception efficiency among mares that remain in production. In spite of the estimated repeatability of the number of cycles needed to achieve a pregnancy being a moderate estimate, it provides nonetheless room for some optimism when retaining or culling mares according to their reproductive performance regarding the number of cycles needed to become pregnant.

In our study, we also took other factors into consideration as potentially affecting the fertility rate, but their effect was not significant, despite a number of studies in the literature reporting different outcomes. The occurrence of endometritis and mare reproductive status are two variables often studied in the literature. Uterine health is paramount for the success of establishing a pregnancy [21]. Endometritis, an inflammatory process of the endometrium, is often described in the literature and is considered to be the most common cause of infertility in mares, as it leads to failure to conceive and early embryonic death [21,22,23]. In fact, it is usually addressed as the most frequent cause of subfertility in mares that cycle normally but do not conceive and in mares that cycle normally and conceive but then suffer early embryonic death [23]. Chronic infection causes a severe, progressive and irreversible fibrotic condition that impairs endometrial function and pregnancy [23]. In our study, the presence of endometritis was not significant, which can be related to the fact that mares with any sign of endometritis were not inseminated without undergoing appropriate treatment. A recent study of our research team has reinforced the importance of performing microbiology and susceptibility tests, even under field conditions, to maximise treatment efficiency and minimise the worldwide problem of antibiotic resistance [24].

Different studies have reported an influence of mare reproductive status on its fertility [1,4,6,25]. A study from 2007 [6], found the highest pregnancy rates to be obtained in maiden mares, then foaling mares, and barren mares were the lowest. Another study states that barren and foaling mares require some sort of uterine therapy more often than young maiden mares, with a clear effect of reproductive status on pregnancy rates [4]. Often barren mares in these studies are defined as those that had been bred the previous year and failed to become pregnant or that have lost their pregnancy [25], and therefore may encompass a group of ‘problem mares’ to conceive and maintain a pregnancy. One possible explanation for the non-significance in our study is that our group of barren mares also includes the ones not deliberately mated previously (for stud farm management decisions). In fact, some authors consider that, occasionally, the gap year between pregnancies is helpful for restoring uterine health.

The findings of this study provide an important benchmark. However, a primary limitation of this study is that the animals in this study were all Lusitano horses residing at the same farm, which introduces inherent selection bias related to specific housing conditions, feeding regimes, and unique management strategies. Consequently, the environmental and management variables are relatively uniform within this sample, and extrapolating these results to the broader Lusitano population or to alternative equine management systems should be done with care. Future studies involving other populations and diverse conditions are required to validate these findings on a global scale.

## 5. Conclusions

Success in AI breeding programmes is limited by different factors. Increasing mare and stallion age produced a marked deleterious effect on fertility. Findings in this study should allow all involved in equine reproduction to make informed and reasoned decisions in order to achieve the best possible reproductive performance in a stud farm, particularly providing benchmark information against which the Lusitano industry can guide its management of mares at insemination.

## Figures and Tables

**Table 1:** Distribution of the number of mares and the number of oestrous cycles per year and per month.

Year/Month	Number of Mares with Fertility Records (Study 1)	Number of Oestrous Cycles Followed (Study 2)
2003	20	
2004	21	
2005	24	
2006	27	
2007	35	
2008	37	
2009	35	
2010	44	
2011	48	
2012	40	
2013	35	
2016	45	88
2017	58	121
2018	52	84
2019	57	99
2020	56	110
2021	61	121
2022	58	109
2023	54	90
2024	53	89
February	61	109
March	179	226
April	217	282
May	287	237
June	116	57

**Table 2:** Significance of factors considered in binary logit model regression analysis of pregnancy per season (*n* = 860 insemination records in 125 mares).

Effect	F-Statistic	Pr > F
Year of breeding	1.46	0.092
Reproductive status	0.70	0.495
Month of AI	18.04	<0.001
Age of mare	20.60	<0.001
Inbreeding coefficient of mare	0.41	0.521
Age of stallion	6.13	0.013
Inbreeding coefficient of stallion	0.94	0.332

**Table 3:** Odds ratios and estimated means for pregnancy rate by month of artificial insemination in the binary logit model used for regression analysis of pregnancy per season. Odds ratios with different superscripts differ (*p* < 0.05).

	Odds Ratio	Pregnancy Rate	
Month	Estimate	Mean	Std. Error
2	3.3282 ^a^	0.9654	0.07952
3	3.7188 ^a^	0.9763	0.05452
4	3.4041 ^a^	0.9678	0.07321
5	2.1298 ^b^	0.8938	0.2223
6	0.7566 ^c^	0.6806	0.5100

**Table 4:** Regression coefficients of the probability of pregnancy after artificial insemination for the significant (*p* < 0.05) covariates considered in the binary logit model.

Effect	Regression Coefficient	Std. Error
Age of mare (years)	−0.1181	0.0260
Age of stallion (years)	−0.0475	0.0192

**Table 5:** Significance of factors considered in binary logit-model regression analysis of pregnancy diagnosis at 14 days post-AI on the 1st cycle (*n* = 473 insemination records in 98 mares).

Effect	F Value	Pr > F
Year of breeding	1.38	0.205
Reproductive status	1.07	0.343
Month of AI	1.73	0.144
Age of mare	0.78	0.379
Inbreeding coefficient of mare	0.60	0.439
Age of stallion	5.13	0.024
Inbreeding coefficient of stallion	3.50	0.062
Pre-AI endometrial oedema	4.39	0.037
Endometritis	2.30	0.130
Type of semen	1.93	0.165

**Table 6:** Regression coefficients of the probability of pregnancy at 14 d after artificial insemination on the 1st cycle for the significant (*p* < 0.10) covariates considered in the binary logit model.

Effect	Regression Coefficient	Std. Error
Age of stallion (years)	−0.0354	0.0156
Inbreeding coefficient of stallion (%)	−0.0382	0.0204
Pre-AI endometrial oedema ^a^	0.2889	0.1379

^a^ score from 0 (no oedema) to 3 (severe oedema).

**Table 7:** Significance of factors in the analysis of the number of cycles to achieve a pregnancy after artificial insemination in Lusitano horses.

Source	F Value	P(F)
Year of breeding	1.90	0.0506
Month of AI	35.47	<0.0001
Reproductive status	0.88	0.3808
Age of mare	5.24	0.0146
Inbreeding coefficient of mare	4.76	0.0503

**Table 8:** Regression coefficients of number of cycles (b_NC_) needed to achieve a confirmed pregnancy after artificial insemination in Lusitano horses, considering the effect of age and inbreeding coefficient of the mare.

Factor	b_NC_
Age of mare (years)	0.024 ± 0.010
Inbreeding coefficient of mare (%)	−0.017 ± 0.008

## Data Availability

Dataset available on request from the authors. The raw data supporting the conclusions of this article will be made available by the authors on request.

## Abbreviations

The following abbreviations are used in this manuscript:

AI	Artificial insemination
PRBS	Pregnancy rate per breeding season
PR1C	Pregnancy diagnosis at 14 days on the first cycle
